# Evaluating the effectiveness of a digital education programme for Swedish municipal politicians responsible for elder care

**DOI:** 10.1108/JHOM-04-2025-0200

**Published:** 2026-02-02

**Authors:** Ulrika Flädjemark, Per Schubert, Susann Viktoria Porter

**Affiliations:** Department of Urban Studies, Malmö University, Malmö, Sweden; Department of Natural Science, Mathematics and Society, Faculty of Education and Society, Malmö University, Malmö, Sweden; Department of Care Science, Faculty of Health and Society, Malmö University, Malmö, Sweden

**Keywords:** Digital education, Elder care, Municipality politicians, Organisational work environment responsibility

## Abstract

**Purpose:**

The aim of this study was to evaluate the effectiveness of a digital education programme developed for municipality politicians accountable for the Swedish elder care. The programme focused on the work environment for different key professions within elder care and on the municipality politicians' work environment responsibility on an organisational level.

**Design/methodology/approach:**

A longitudinal single-group pre-experimental research design was used to evaluate a digital education programme regarding the organisational and social work environment within elder care. The participants were municipality politicians accountable for the Swedish elder care. The evaluation was based on self-reported responses to a questionnaire – at T1, before the intervention (*N* = 81); at T2, zero to two weeks after the intervention (*n* = 35); and at T3, three months post education (*n* = 35). Longitudinal changes within the intervention group were examined.

**Findings:**

The digital education programme substantially enhanced municipality politicians' self-rated knowledge of professions in both special housing and home care settings. This improvement was observed immediately after the programme at T2 and was either maintained or further increased at the three-month follow-up at T3. The absence of clear differences between T2 and T3 indicates that the knowledge gains were sustained over this period.

**Practical implications:**

The results suggest that a digital education programme targeting the organisational level of the municipality-driven elder care, that is, accountable politicians, can help improve the understanding of work environment management regarding elder care, and thereby be a way to address challenges in elder care employee situations.

**Originality/value:**

The focus is on addressing the organisational level instead of first-line managers and/or individuals.

## Introduction

This article evaluates the effectiveness of a digital education programme aimed at enhancing work environment knowledge. The programme specifically targets municipality politicians in Sweden, who, in their role as employer representatives, have responsibility for the work environment within elder care – an area managed at the municipal level. The study focuses on examining the effectiveness of the programme in improving this knowledge among politicians at the organisational level.

Research on work environment conditions in the public sector, that is, politically governed healthcare settings, is plentiful. A systematic literature review from the Nordic countries between 2016 and 2022 on the matter of work environment conditions emphasises organisational risk and health-promoting factors (Swedish Agency for Work Environment Expertise, 2023). The review concludes that the research typically concerns workplace and individual levels and the importance of balancing demands and resources (Bakker and Demerouti, 2017; Corin and Björk, 2016; Xanthopoulou *et al.*, 2007a). The latter meaning that job resources interact with, and to some extent buffer the impact of job demands and may hence hinder the development of health problems deriving from a poor social and organisational work environment (Dollard and Bakker, 2010). The organisational level, on the other hand, defined “as the level at which decisions are made about the organisation's structure, principles for work or production, and the values that should underpin the work” (Swedish Agency for Work Environment Expertise, 2023, p. 7), is much less researched.

For nearly 50 years, employers in Sweden have had comprehensive work environment responsibility for the employees engaged in their organisations. The Swedish Work Environment Act (SWA) of 1977, together with provisions issued by the Swedish Work Environment Agency (SWEA), specifies that employers have a legal responsibility for all aspects of the work environment related to the duties of their employees. Thus, employers are required to identify and eliminate or alleviate risks in every aspect of the work environment; physical, social and organisational that could lead to ill health (SFS, 1977:1160 Swedish Work Environment Act, 1977; Ahlberg, 2018; Gillberg, 2018).

As politicians represent the municipality as employers, they have the ultimate responsibility for the work environments of the various professions within their area of accountability (Swedish Association of Local Authorities and Regions, 2022). Sweden has approximately 34,500 elected politicians in its 290 municipalities, of these 96% are part-time politicians (Statistics Sweden, 2024). How many of these municipality politicians are elected to be on an elder board is not clear due to that the municipality councils independently select the boards and committees that govern the various areas of responsibility, and therefore, board and committee structures can differ between municipalities. Although a political board chair has specific obligations to convene board meetings, conduct the meetings and prepare minutes, the board chairs and the board members each have a single vote and are equally responsible for decisions made (SFS, 2017:725 Local Government Act, 2017).

As municipality civil servants often make operational decisions according to delegations from the municipality boards, holding a position in these boards entails the obligation to ensure that the SWA is adhered to. The responsibility is equally shared amongst board chairs and board members (SFS, 2017:725 Local Government Act, 2017; Swedish Association of Local Authorities and Regions, 2022), and it is, consequently, important for all board chairs and board members to be aware of their employer responsibility. Regarding this responsibility, the evaluated effectiveness of this digital education programme addresses two aspects.

Firstly, it is fundamental for those who are responsible for the work environment to have knowledge of the work environmental legislation. The SWA and the provisions regarding work environmental aspects, for example, Organisational and Social Work Environment (AFS, 2015:4, 2015) and Systematic Work Environment Management (AFS, 2001:1, 2001), are mandatory knowledge and cannot be neglected. Even if the day-to-day efforts concerning work environment at individual and workplace levels are delegated, the ultimate responsibility to manage viable goal setting and overarching organisational objectives cannot be fully delegated. Accountable management on an organisational level must, for example, be aware of the principles of Systematic Work Environment Management (AFS, 2001:1, 2001), and on a yearly basis investigate all work environmental settings, risk assess what has been found and analyse the findings, and finally take the necessary measures and follow up on whether to remove or mitigate the assessed risks. Furthermore, it is beneficial for the accountable management to be aware of the work environmental research that has formed the basis for the regulations and that explains why certain aspects are considered crucial for establishing a good work environment. With knowledge, informed decisions may be executed and relevant work environment management carried out effectively.

Secondly, the significance of understanding the actual work done by professions in an elder care context should be considered, as should the specific circumstances in elder care work situations. High sick leave rates (Swedish Social Insurance Agency, 2024), staff leaving, often due to a deficient social and organisational work environment (Swedish Municipal Workers' Union, 2023), and staff shortages, especially future wise, as a result of changing population demographics with more elderly individuals needing special housing or home care (National Board of Health and Welfare, 2023), combined with staff leaving due to retirement, generates a challenge (Swedish Work Environment Authority, 2024; Szebehely *et al.*, 2017).

### Earlier research

Earlier research on municipality politicians' responsibility for Swedish elder care indicates that politicians may lack knowledge of their responsibility for the work environment as employers, which also extends to knowledge of the work performed by different professions in this context (Porter *et al*., 2025; Porter, 2022; Porter and Muhonen, 2021). Although previous research has demonstrated a substantial body of knowledge regarding the work environment, there remains a recognised need to enhance such knowledge at the organisational level (Swedish Agency for Work Environment Expertise, 2023). Since earlier research also display a motivation among politicians to enhance their own knowledge of their area of responsibility (Porter, 2022), it is of interest to investigate how actual work environment knowledge, if deficient, could be enhanced within this group.

Hence, this study investigates whether a digital educational programme targeted at organisational level may serve to improve work environment knowledge. Both the aspects of work environmental legislation and elder care work specifics are addressed. The aim of this study was to evaluate the effect of a digital educational programme developed for municipality politicians accountable for the Swedish elder care. Longitudinal self-rated knowledge changes within the group were examined. We hypothesised that there would be changes in self-rated knowledge after the intervention.

## Materials and methods

### Research design

This study used a single-group pre-experimental research design (Reichardt, 2002) measuring self-assessed knowledge before the digital education programme (T1), and at two points after the programme: zero to two weeks (T2) and three months after (T3). The participants agreed to participate in the study by providing their written consent and accessed a questionnaire and the digital education programme through two links sent by email links. Data collection began in January 2024 and was concluded in October 2024. The present study was part of a larger project exploring the knowledge of the organisation and social work environment among municipality politicians accountable for the Swedish elder care (Porter and Flädjemark, submitted).

Previous research has shown that Swedish politicians accountable for elder care may lack relevant knowledge while at the same time expressing a desire to learn more about the organisation and social work environment within elder care (Porter and Muhonen, 2021; Porter, 2022). To attempt to address this deficit, a digital education programme was developed through a collaboration with the authors of the present study along with four other researchers from three Swedish universities –Stockholm University, Lund University and Malmö University.

### Digital education programme

The educational programme was divided into two parts, here described as module 1 and module 2. Module 1 comprised six digital recorded lectures, 15–22 min each, regarding the organisational and social work environment and covered the following: 1. *The organisational and social work.* 2. *The Work Environment Act, and employers' responsibility.* 3. *Mental illness.* 4. *Employment conditions and working conditions in a changing care for the elderly. What does it take for the staff to have the energy and the desire to stay in their jobs?* 5. *Managing differences. Ethical diversity, the multi-ethnic working groups of elderly care in everyday practice.* 6. *Preventing and managing mental illness in the workplace, the leadership role and balancing demands and control*.

Module 2 focused on the work environments of the key professions in elder care and was developed through focus-group and individual in-depth interviews, to gain an insight into how the different professionals (i.e., care assistant, assistant nurse, registered nurse, physiotherapist, occupational therapist and first-line manager, within both special housing and home care settings) experience their work environment (Table 1). The corresponding union organisations for each profession were contacted to assist with recruitment. Due to the difficulty in recruiting participants among the physiotherapist and first-line manager professions, in-depth interviews with open-ended questions were performed individually with participants from each profession in special housing and home care (Table 1).

**Table 1:** Characteristics of the participants in focus group and individual in-depth interviews for development of module 2

Characteristics	*n*	Special housing	Home care	Female/male	Age in years (mean/range)	Years in profession (mean/range)
Focus group interviews (*n* = 4)						
Care assistants	11	5	6	10/1	48.2/31–64	24.7/6–39
Assistant nurses	7	2	5	5/2	47.6/35–64	21.6/6–45
Registered nurses	6	3	3	5/1	54.7/40–64	14.5/6–24
Occupational therapists	4	2	2	3/1	42.0/26–54	8.8/1–23
Individual in-depth interviews (*n* = 16)						
Care assistants	2	1	1	2/0	52.0/46–58	31.0/28–34
Assistant nurses	2	1	1	1/1	37.5/28–47	14.0/3–25
Registered nurses	2	1	1	2/0	40.0/33–47	6.0/6–6
Physiotherapists	4	2	2	3/0	46.3/34–56	11.0/4–20
Occupational therapists	3	2	1	3/0	49.3/45–54	20.2/21–20
First-line managers	4	1	3	4/0	48.8/32–56	8.0/2–12

All interviews (focus group and individual) were transcribed verbatim and analysed using thematic content analysis (Braun and Clarke, 2006). The results where then summarised by a media producer from the communication department at Malmö University with substantial experience of transforming qualitative research results into abbreviated manuscripts, in collaboration with the research team. Subsequently, the manuscripts were voice recorded by students from the drama programme at Malmö University, and photographic images were added to the voice recordings, illustrating the dialogues. The photographs were taken by the media producer and the project leader, from a wide range of contexts, to illustrate the content of the voice recordings. In total, 16 films were produced and included in module 2. The average duration of the films was 3 ½ minutes. Two films described the working environment for care assistants and assistant nurses, one from the context of special housing and one from home care. Due to the extensive material, three films were produced regarding registered nurses, physiotherapists, occupational therapists and first-line managers. Verification of the films' authenticity was sought from the professionals within the respective union organisations. The verification groups watched the final versions of the films involving their professionals' groups and provided feedback to the project leader. No changes were made, as the verification groups considered the films to be accurate.

## Data collection and longitudinal evaluation

A web-based application, REDCap (REDCap, n.d.), was used to create and distribute a questionnaire to the participants. Baseline characteristics were collected at T1 and comprised demographic data regarding gender, age, educational level, political affiliation, years as a responsible politician for elder care, as well as a question regarding work environment training prior to participation in the present study. The investigative questionnaire was divided into two parts. Part 1 was collected at T1, T2 and T3 and included self-rated knowledge levels regarding the work of the key professions in elder care, that is, care assistant, assistant nurse, registered nurse, physiotherapist, occupational therapist and first-line manager, within both special housing and home care settings. The question included was *“How do you grade your knowledge of the work of the following professions?”* [(1) *Care assistant within special housing; Care assistant within home care;* (2) *Assistant nurse within special housing; Assistant nurse within home care;* (3) *Registered nurse within special housing; Registered nurse within home care;* (4) *Physiotherapist within special housing; Physiotherapist within home care;* (5) *Occupational therapist within special housing; Occupational therapist within home care;* (6) *First-line manager within special housing; First-line manager within home care*], and the participants were asked to grade their levels of knowledge as *Very high, Fairly high, Rather low, Very low* or *Do not know.*

Part 2 was collected at T1, T2 and T3 and included self-rated knowledge levels regarding accountability for work environments in elder care: (1) *I have accountability for the work environment of the elder care staff;* (2) *According to the Work Environment Act, I have accountability as an employer for the staff's work environment in elder care;* (3) *I know what can cause mental health problems among staff in elder care;* (4) *I know what can promote a healthy work environment in elder care.* The politicians were asked to grade their levels of knowledge as *Very high*, *Fairly high*, *Rather low*, *Very low* or *Not at all*. Note that statement 1 was only included in T1, and therefore, no differences regarding this statement between T1, T2 and T3 are included in this study.

When the questionnaire and modules 1 and 2 were developed, feedback was received from the research Centre for Work Life Studies (CTA) to which the project leader (last author) and first author were affiliated, along with the union organisations of the key professions included in module 2. Feedback was also received from three politicians who did not participate in the intervention study. These politicians were recruited from previous studies (Porter and Muhonen, 2021; Porter, 2022) and represented the three largest Swedish political parties at the time of the evaluation in 2024, that is, the Social Democrats, the Moderates and the Swedish Democrats. They all had the chair role on their respective board. All the participants who were recruited to give feedback, watched the digital education programme and tested the questionnaire. Based on the feedback from the CTA, minor changes were made on the order of the films within module 1.

### Participants in the intervention study

Participants eligible for the study were politicians elected to a municipality board responsible for elder care, who were 18 years or above and understood oral and written Swedish. All board chairs responsible for elder care across Sweden's 290 municipalities were contacted by the project leader, who emailed them information about the study and a consent form. The board chairs were encouraged to forward the invitation to their board members. Due to difficulties in recruiting, information of the study was also emailed to board vice-chairs, deputy board vice-chairs, board members and deputy board members on elder care boards.

### Ethical considerations

This study was conducted in accordance with the ethical guidelines of the *Declaration of Helsinki – Ethical Principles for Medical Research Involving Human Subjects* (World Medical Association, 2001). In developing module 2, written and verbal information concerning the objective of the study was given to potential participants, and a signed consent form was received from all the participants who accepted participation in the interviews. Regarding the intervention study, an email with information about the study and the objective of the evaluation of the digital education programme was sent to all potential participants, along with a consent form. Study participants then emailed or posted a signed written consent form to the project leader. All emailed consent forms were printed out and stored in a locked safe box, and the original emails were deleted. The rights to terminate participation and confidentiality were guaranteed. Ethical approvals were sought and granted by the Regional Ethical Board in Linköping, Sweden (Reg. no. 2022-01999-01), before the development of module 2 and before the intervention study began.

### Statistical methods

The collected questionnaire data were exported from REDCap into SPSS Statistics Version 30 for analysis. Baseline characteristics were compiled into tables to show the characteristics of the participating municipality politicians (Table 2). To evaluate the effects of the intervention, the Wilcoxon signed-rank test (Blom and Holmquist, 1998) was conducted on the differences between pre-intervention and post-intervention scores, that is, the scores between T1 and T2, T1 and T3 and T2 and T3 of the municipality politicians' self-rated knowledge. In total, 45 tests were performed, including 18 for self-rated knowledge levels about key professions in special housing, 18 for self-rated knowledge levels about key professions in home care and 9 for self-rated knowledge levels about accountability. Two-sided hypotheses were tested stating differences between T1 and T2, T1 and T3 and T2 and T3 for each profession and each accountability variable. The calculations of *p*-values were based on the corresponding null hypotheses stating that there were no differences, and if a *p*-value was lower than or equal to 0.05, then the null hypothesis was rejected, and the difference was considered statistically significant.

**Table 2:** Characteristics of the participating municipality politicians at T1, T2 and T3

Characteristics	*n*	%	Years (mean and range)
Age	80/34/34	98.77/97.14/97.14	60.3 & 33–91/60.7 & 33–91/61.5 & 33–80
*Missing value*	1/1/1	1.23/2.86/2.86	
Female and male	51 & 30/26 & 9/28 & 7	62.96 & 37.04/74.29 & 25.71/80.00 & 20.00	
*Geographic region of Sweden*			
Götaland (south)	37/12/14	45.68/34.29/40.00	
Svealand (mid-)	35/18/17	43.21/51.43/48.57	
Norrland (north)	8/4/4	9.88/11.43/11.43	
*Missing value*	1/1/0	1.23/2.86/0.00	
*Educational level*			
Upper secondary	22/11/10	27.16/31.43/28.57	
University	59/24/25	72.84/68.57/71.43	
*Political party*			
The Moderates	26/5/10	32.10/14.29/28.57	
The Social Democrats	20/12/12	24.69/34.29/34.29	
The Christian Democrats	9/1/2	11.11/2.86/5.71	
The Left Party	11/8/6	13.58/22.86/17.14	
The Centre Party	4/1/0	4.94/2.86/0.00	
The Liberals	3/2/1	3.70/5.71/2.86	
The Swedish Democrats	3/2/1	3.70/5.71/2.86	
The Green Party	2/1/0	2.47/2.86/0.00	
Local independent parties	3/3/3	3.70/8.57/8.57	
*Political assignment for elder care*			
Board chair	23/11/12	28.40/31.43/34.29	
Board vice-chair	12/6/6	14.81/17.14/17.14	
Deputy board vice-chair	10/2/4	12.35/5.71/11.43	
Board member	23/11/8	28.40/31.43/22.86	
Deputy board member	12/4/5	14.81/11.43/14.29	
*Missing value*	1/1/0	1.23/2.86/0.00	
*Years as responsible politician for*			
elder care	76/34/34	93.83/97.14/97.14	4.7 & 1–30/4.6 & 1–22/4.8 & 1–30
*Missing value*	5/1/1	6.17/2.86/2.86	
*Work environment training received during the last two years*			
Yes	18/10/9	22.22/28.57/25.71	
No	63/25/26	77.78/71.43/74.29	

The test results were compiled into tables to show the *p*-values, *z*-values, *r* values, *n* total, *n* positive differences, *n* negative differences and *n* ties. The effect size (*r*) was calculated as the *z*-value divided by the square root of the total number of pairs *n* and interpreted using Cohen's criteria, where 0.10 ≤ *r* < 0.3 is a small effect size, 0.30 ≤ *r* < 0.50 is a medium effect size and *r* ≥ 0.50 is a large effect size (Fritz *et al.*, 2012). The tables were complemented with bar charts to show percentage distributions of difference magnitudes between T1 and T2 for the key profession variables in special housing and also for the accountability variables. The difference magnitudes are the stepwise differences between T1 and T2 scores on the ordinal scales, for example, a difference magnitude of 2 between the scores Rather low (T1) and Very high (T2). Bar charts were included for all municipality politicians and also for chairs (board chairs, board vice-chairs and deputy board vice-chairs) and members (board members and deputy board members), to identify differences between the groups. Bar charts were only included for special housing, as the charts for home care were very similar, the reasoning about special housing therefore being applicable to home care, too.

## Results

### Baseline characteristics

At T1 (*N* = 81), the politicians accountable for elder care had roles distributed across 23 board chairs, 12 board vice-chairs, 10 deputy board vice-chairs, 23 board members and 12 deputy board members (1 participant did not specify their board assignment) (Table 2). There were 51 women and 30 men with a mean age of 60.3 years (range 33–91 years). The mean time as a responsible politician for elder care was 4.7 years (range 1–30 years). Participants were geographically distributed across Sweden's three regions: the southern region of Götaland (*n* *= *37), the central region of Svealand (*n* *= *35) and the northern region of Norrland (*n = *8). Approximately 73% of the participants (*n = *59) had a university level education and about 27% (*n = *27) had an upper-secondary education. Twenty-six participants belonged to the largest right-wing political party (The Moderates) and 20 belonged to the largest left-wing political party (The Social Democrats). The remaining 35 participants were distributed between other political parties (The Christian Democrats [*n = *9], The Left Party [*n = *11], The Centre Party [*n = *4], The Liberals [*n = *3], The Swedish Democrats [*n = *3], The Green Party [*n = *2] and local independent parties [*n = *3]). Additional participant information is shown in Table 2, including characteristics for the participants at T2 and T3. In addition, occupational status was represented by about 40 different professions, including accountant, administrator, registered nurse, engineer, full-time politician, manager, sociologist, teacher and welder. Twenty-two participants were retired.

### Longitudinal changes in self-rated knowledge of the work of the key professions

**Figure 1:**
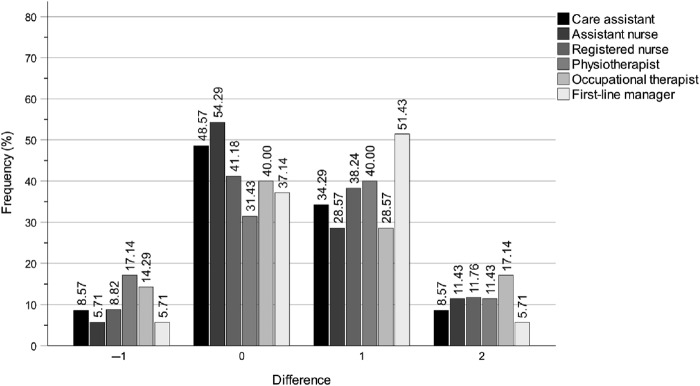
Digital education programme effects for municipality chair and member politicians between T1 and T2 on self-rated knowledge levels of the work of the key professions in special housing. Note: The magnitude differences on the *x*-axis show the stepwise differences between T1 and T2 scores on the scale Do not know > Very low > Rather low > Fairly high > Very high. Source: Authors’ own work

**Figure 2:**
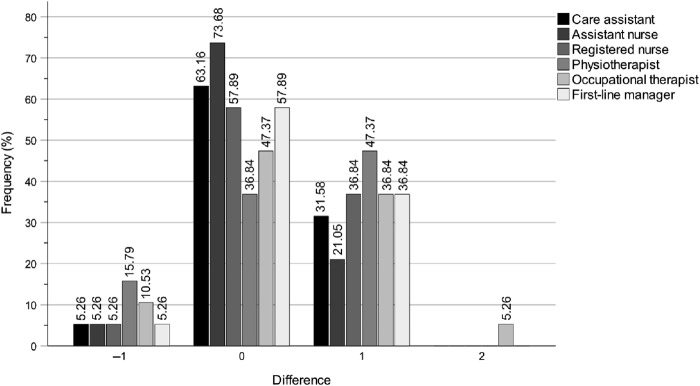
Digital education programme effects for municipality *chair* politicians between T1 and T2 on self-rated knowledge levels of the work of the key professions in special housing. Note: The magnitude differences on the *x*-axis show the stepwise differences between T1 and T2 scores on the scale Do not know > Very low > Rather low > Fairly high > Very high. Source: Authors’ own work

**Figure 3:**
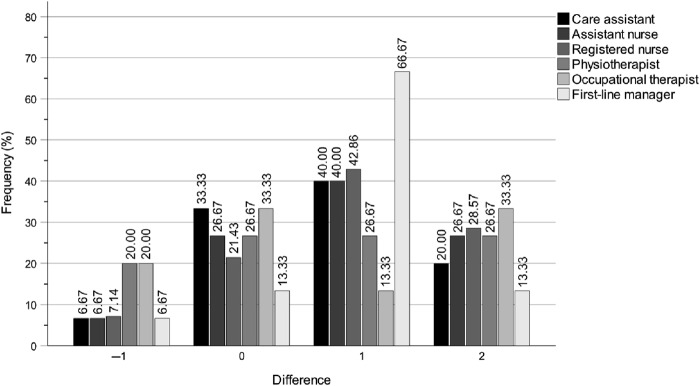
Digital education programme effects for municipality *member* politicians between T1 and T2 on self-rated knowledge levels of the work of the key professions in special housing. Note: The magnitude differences on the *x*-axis show the stepwise differences between T1 and T2 scores on the scale Do not know > Very low > Rather low > Fairly high > Very high. Source: Authors’ own work

The analysis demonstrated that the digital education programme substantially enhanced the municipality politicians' self-rated knowledge levels of the work of the key professions in both special housing and home care settings. This is evident from the *p*-values, *z*-values, *r*-values, *n* totals, *n* positive differences, *n* negative differences and *n* ties presented in Table 3. Note that the *r*-value not only reflects the direction of a participant's change between T1 and T2, as well as T1 and T3, but also the magnitude of that change. Consequently, the *r*-value at T3 may increase or decrease independently of the number of participants who improved, declined or remained stable. The improvements were observed immediately after the education programme at T2 and were either largely maintained or further increased until the three-month follow-up at T3. Notably, there were no clear differences between T2 and T3, which indicates that the effects were largely sustained over this period. Figures 1–3 show the percentage distributions of difference magnitudes between T1 and T2 in special housing, for all municipality politicians and for chairs and members separately. The values on the *x*-axis represent the stepwise differences between T1 and T2 values on the ordinal scale *Do not know > Very low > Rather low > Fairly high* > *Very high*. By comparing the distributions for all municipality politicians (Figure 1) with chairs (Figure 2) and members (Figure 3), it is clear that all groups had enhanced their self-rated knowledge, but also that the most pronounced improvement is within the member group. As noted in the method section, the similarity with the bar charts for home care indicates that these results are equally applicable to home care.

**Table 3:** Digital education programme effects for municipality chair and member politicians on self-rated knowledge levels of the work of the key professions in special housing and home care

Housing	T1 and T2	T1 and T3	T2 and T3
*Care assistant*			
SH	0.004*/2.878/0.49/35/15/3/17	0.005*/2.829/0.49/35/13/2/20	1.000/0.000/0.00/26/4/4/18
HC	0.012*/2.517/0.43/35/11/2/22	0.012*/2.517/0.43/35/11/2/22	1.000/0.000/0.00/26/4/4/18
*Assistant nurse*			
SH	0.003*/2.996/0.51/35/14/2/19	0.002*/3.120/0.53/35/15/2/18	1.000/0.000/0.00/26/3/3/20
HC	0.017*/2.389/0.41/34/10/2/22	0.008*/2.668/0.45/35/12/2/21	0.655/0.447/0.09/26/3/2/21
*Registered nurse*			
SH	0.002*/3.164/0.54/34/17/3/14	<0.001*/3.477/0.59/35/16/1/18	0.705/-0.378/-0.07/26/3/4/19
HC	0.001*/3.220/0.54/35/14/1/20	0.002*/3.119/0.53/35/15/2/18	0.705/-0.378/-0.07/26/3/4/19
*Physiotherapist*			
SH	0.007*/2.675/0.45/35/18/6/11	0.045*/2.008/0.34/35/18/6/11	0.564/-0.577/-0.11/26/5/7/14
HC	0.005*/2.805/0.47/35/17/5/13	0.023*/2.274/0.38/35/19/6/10	1.000/0.000/0.00/26/5/5/16
*Occupational therapist*			
SH	0.006*/2.751/0.46/35/16/5/14	0.001*/3.201/0.54/35/19/4/12	0.782/0.277/0.05/26/7/6/13
HC	0.003*/2.954/0.51/34/17/4/13	<0.001*/3.539/0.61/34/20/3/11	0.564/0.577/0.11/26/7/5/14
*First-line manager*			
SH	<0.001*/3.772/0.63/35/20/2/13	<0.001*/3.392/0.57/35/17/2/16	0.655/-0.447/-0.08/26/2/3/21
HC	<0.001*/3.752/0.63/35/20/2/13	0.001*/3.258/0.55/35/16/2/17	0.705/-0.378/-0.07/26/3/4/19

**Note(s):** *Significant at the 0.05 level. The Wilcoxon Signed Rank Test results for the digital education programme effects between T1 and T2, T1 and T3 and T2 and T3, in both the contexts of Special Housing (SH) and Home Care (HC). The number sequences in the table show *p*-value/*z*-value/*r*-value/*n* total/*n* positive differences/*n* negative differences/*n* ties

Overall, the most notable results were the substantial self-rated knowledge increases regarding the work of registered nurses, occupational therapists and first-line managers, in both special housing and home care settings, between T1 and T2, and T1 and T3 (Table 3 and Figure 1). The intervention effects were not only immediate for all three professions at T2 but also sustained or further increased for registered nurses and occupational therapists up to three months post-intervention at T3. The effects for first-line managers showed a decrease at T3 compared to T2. Even though the effects were not equally strong for care assistants, assistant nurses and physiotherapists they were still quite clear, although the intervention effect at T3 showed a decrease for physiotherapists.

### Longitudinal changes in self-rated knowledge of the work of care assistants

Between T1 and T2, the digital education programme improved the municipality politicians' self-rated knowledge of the work of care assistants in special housing (Table 3 and Figure 1). Specifically, 15 participants demonstrated an increase, 3 exhibited a decrease and 17 showed no change (*p* = 0.004, *z* = 2.878, *r* = 0.49). Based on the effect size (*r*), this improvement was sustained between T1 and T3, with 13 participants showing an increase, 2 showing a decrease and 20 showing no change (*p* = 0.005, *z* = 2.829, *r* = 0.49).

The intervention also had a positive effect between T1 and T2 on the politicians' self-rated knowledge of the work of care assistants in home care, with 11 participants showing an increase, 2 showing a decrease and 22 showing no change (*p* = 0.012, *z* = 2.517, *r* = 0.43) (Table 3). This improvement was sustained between T1 and T3, with 11 participants showing an increase, 2 showing a decrease and 22 showing no change (*p* = 0.012, *z* = 2.517, *r* = 0.43).

### Longitudinal changes in self-rated knowledge of the work of assistant nurses

Between T1 and T2, the education programme substantially improved the municipality politicians' self-rated knowledge of the work of assistant nurses in special housing (Table 3 and Figure 1). Specifically, 14 participants demonstrated an increase, 2 exhibited a decrease, and 19 showed no change (*p* = 0.003, *z* = 2.996, *r* = 0.51). This improvement continued between T1 and T3, with 15 participants showing an increase, 2 showing a decrease and 18 showing no change (*p* = 0.002, *z* = 3.120, *r* = 0.53).

The intervention also had a positive effect between T1 and T2 on the politicians' self-rated knowledge of the work of assistant nurses in home care, with 10 participants showing an increase, 2 showing a decrease and 22 showing no change (*p* = 0.017, *z* = 2.389, *r* = 0.41) (Table 3). This improvement continued between T1 and T3, with 12 participants showing an increase, 2 showing a decrease and 21 showing no change (*p* = 0.008, *z* = 2.668, *r* = 0.45).

### Longitudinal changes in self-rated knowledge of the work of registered nurses

Between T1 and T2, the education programme substantially improved the municipality politicians' self-rated knowledge of the work of registered nurses in special housing (Table 3 and Figure 1). Specifically, 17 participants demonstrated an increase, 3 exhibited a decrease and 14 showed no change (*p* = 0.002, *z* = 3.164, *r* = 0.54). This improvement continued between T1 and T3, with 16 participants showing an increase, 1 showing a decrease and 18 showing no change (*p* < 0.001, *z* = 3.477, *r* = 0.59).

The intervention also had a considerable effect between T1 and T2 on the politicians' self-rated knowledge of the work of registered nurses in home care, with 14 participants showing an increase, 1 showing a decrease and 20 showing no change (*p* = 0.001, *z* = 3.220, *r* = 0.54) (Table 3). This improvement was largely sustained between T1 and T3, with 15 participants showing an increase, 2 showing a decrease and 18 showing no change (*p* = 0.002, *z* = 3.119, *r* = 0.53).

### Longitudinal changes in self-rated knowledge of the work of physiotherapists

Between T1 and T2, the education programme improved the municipality politicians' self-rated knowledge of the work of physiotherapists in special housing (Table 3 and Figure 1). Specifically, 18 participants demonstrated an increase, 6 exhibited a decrease and 11 showed no change (*p* = 0.007, *z* = 2.675, *r* = 0.45). This improvement decreased between T1 and T3, with 18 participants showing an increase, 6 showing a decrease and 11 showing no change (*p* = 0.045, *z* = 2.008, *r* = 0.34).

The intervention also had a positive effect between T1 and T2 on the politicians' self-rated knowledge of the work of physiotherapists in home care, with 17 participants showing an increase, 5 showing a decrease and 13 showing no change (*p* = 0.005, *z* = 2.805, *r* = 0.47) (Table 3). This improvement decreased between T1 and T3, with 19 participants showing an increase, 6 showing a decrease and 10 showing no change (*p* = 0.023, *z* = 2.274, *r* = 0.38).

### Longitudinal changes in self-rated knowledge of the work of occupational therapists

Between T1 and T2, the education programme improved the municipality politicians' self-rated knowledge of the work of occupational therapists in special housing (Table 3 and Figure 1). Specifically, 16 participants demonstrated an increase, 5 exhibited a decrease and 14 showed no change (*p* = 0.006, *z* = 2.751, *r* = 0.46). This improvement continued considerably between T1 and T3, with 19 participants showing an increase, 4 showing a decrease and 12 showing no change (*p* = 0.001, *z* = 3.201, *r* = 0.54).

The intervention had a considerable effect between T1 and T2 on the politicians' self-rated knowledge of the work of occupational therapists in home care, with 17 participants showing an increase, 4 showing a decrease and 13 showing no change (*p* = 0.003, *z* = 2.954, *r* = 0.51) (Table 3). This improvement continued considerably between T1 and T3, with 20 participants showing an increase, 3 showing a decrease and 11 showing no change (*p* < 0.001, *z* = 3.539, *r* = 0.61).

### Longitudinal changes in self-rated knowledge of the work of first-line managers

Between T1 and T2, the education programme substantially improved the municipality politicians' self-rated knowledge of the work of first-line managers in special housing (Table 3 and Figure 1). Specifically, 20 participants demonstrated an increase, 2 exhibited a decrease and 13 showed no change (*p* < 0.001, *z* = 3.772, *r* = 0.63). This improvement decreased between T1 and T3, although remaining on a high level, with 17 participants showing an increase, 2 showing a decrease and 16 showing no change (*p* < 0.001, *z* = 3.392, *r* = 0.57).

The intervention also had a considerable effect between T1 and T2 on the politicians' self-rated knowledge of the work of first-line managers in home care, with 20 participants showing an increase, 2 showing a decrease and 13 showing no change (*p* < 0.001, *z* = 3.752, *r* = 0.63) (Table 3). This improvement decreased between T1 and T3, although remaining on a high level, with 16 participants showing an increase, 2 showing a decrease and 17 showing no change (*p* = 0.001, *z* = 3.258, *r* = 0.55).

### Longitudinal changes in self-rated knowledge of accountability for the work environment

Similar to the digital education programme's effects on self-rated knowledge levels of the work of the key professions, it was clear that the intervention substantially enhanced the municipality politicians' self-rated knowledge levels of accountability for the work environment. This is evident from the *p*-values, *z*-values, *r*-values, *n* totals, *n* positive differences, *n* negative differences and *n* ties presented in Table 4. These improvements were gained at T2 and then largely sustained or even increased until the three-month follow-up at T3, although the intervention effect at T3 showed a slight decrease for legal responsibility. Notably, there were no clear differences between T2 and T3, which indicates that the effects were largely sustained over this period.

**Table 4:** Digital education programme effects for municipality chair and member politicians on self-rated knowledge levels of accountability for the work environments of the key professions in elder care

T1 and T2	T1 and T3	T2 and T3
*Legally responsible for work environment*		
0.004*/2.869/0.48/35/11/1/23	0.023*/2.275/0.40/33/9/3/21	0.564/-0.577/-0.11/26/1/2/23
*Knowledge of causes of mental illness*		
0.001*/3.207/0.54/35/14/1/20	0.002*/3.082/0.53/34/13/1/20	0.763/0.302/0.06/26/6/5/15
*Knowledge of how to promote healthy workplaces*		
0.012*/2.502/0.44/33/14/4/15	0.001*/3.214/0.55/34/14/1/19	0.157/1.414/0.28/25/6/2/17

**Note(s):** *Significant at the 0.05 level. The Wilcoxon Signed Rank Test results for the digital education programme effects between T1 and T2, T1 and T3 and T2 and T3. The number sequences in the table show *p*-value//*z*-value/*r*-value/*n* total/*n* positive differences/*n* negative differences/*n* ties

**Figure 4:**
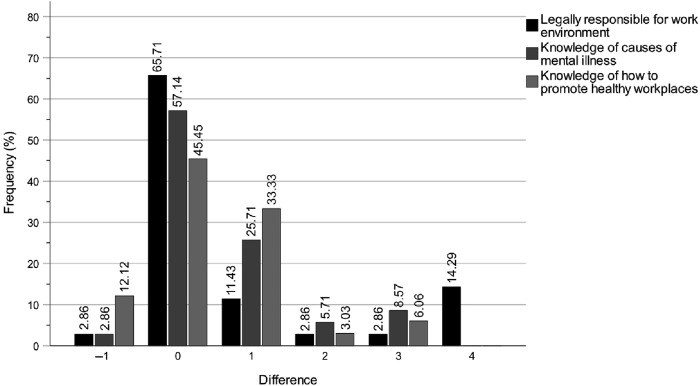
Digital education programme effects for municipality chair and member politicians between T1 and T2 on self-rated knowledge levels of accountability for the work environments of the key professions in elder care. Note: The magnitude differences on the *x*-axis show the stepwise differences between the T1 and T2 scores on the scale Not at all > Very low > Rather low > Fairly high > Very high. Source: Authors’ own work

**Figure 5:**
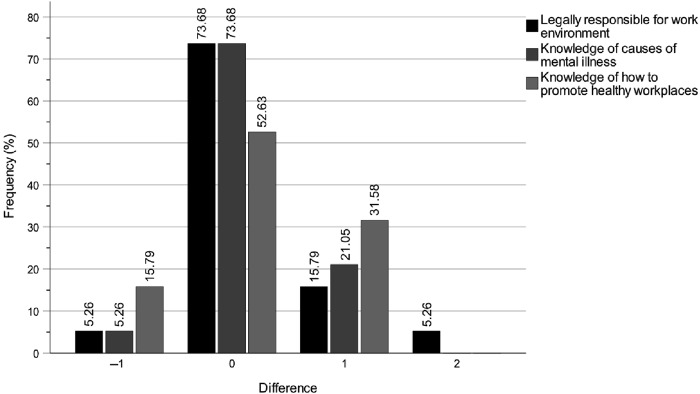
Digital education programme effects for municipality *chair* politicians between T1 and T2 on self-rated knowledge levels of accountability for the work environments of the key professions in elder care. Note: The magnitude differences on the x-axis show the stepwise differences between the T1 and T2 scores on the scale Not at all > Very low > Rather low > Fairly high > Very high. Source: Authors’ own work

**Figure 6:**
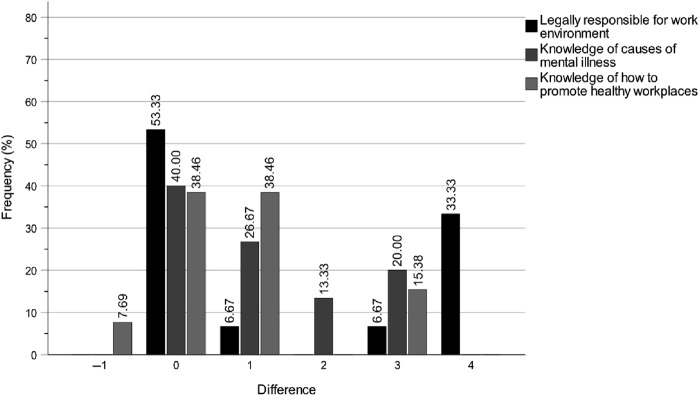
Digital education programme effects for municipality *member* politicians between T1 and T2 on self-rated knowledge levels of accountability for the work environments of the key professions in elder care. Note: The magnitude differences on the x-axis show the stepwise differences between the T1 and T2 scores on the scale Not at all > Very low > Rather low > Fairly high > Very high. Source: Authors’ own work

Figures 4–6 show the percentage distributions of difference magnitudes between T1 and T2 in special housing, for all municipality politicians and for chairs and board members. The difference values on the *x*-axis show the stepwise differences between T1 and T2 values on the scale Not at all > Very low > Rather low > Fairly high > Very high. Like the intervention effects on self-rated knowledge levels of the work of the key professions, by comparing the distributions for all municipality politicians (Figure 4) with chairs (Figure 5) and members (Figure 6), it is not only clear that all groups have improved but also that the most pronounced improvement is within the member group. As noted in the method section, the similarity of the bar charts for home care indicates that these results are equally applicable to home care.

### Longitudinal changes in self-rated knowledge of legal responsibility for the work environment

Between T1 and T2, the intervention improved the municipality politicians' self-rated knowledge of legal responsibility for the work environment (Table 4 and Figure 4). Specifically, 11 participants demonstrated an increase, 1 participant exhibited a decrease and 23 participants showed no change (*p* = 0.004, *z* = 2.869, *r* = 0.48). Based on the effect size (*r*), this improvement decreased between T1 and T3, with 9 participants showing an increase, 3 participants showing a decrease and 21 participants showing no change (*p* = 0.023, *z* = 2.275, *r* = 0.40).

### Longitudinal changes in self-rated knowledge of causes of mental illness

Between T1 and T2, the intervention substantially improved the municipality politicians' self-rated knowledge of causes of mental illness (Table 4 and Figure 4). Specifically, 14 participants demonstrated an increase, 1 participant exhibited a decrease and 20 participants showed no change (*p* = 0.001, *z* = 3.207, *r* = 0.54). This improvement was largely sustained between T1 and T3, with 13 participants showing an increase, 1 participant showing a decrease and 20 participants showing no change (*p* = 0.002, *z* = 3.082, *r* = 0.53).

### Longitudinal changes in self-rated knowledge of how to promote healthy workplaces

Between T1 and T2, the intervention improved the municipality politicians' self-rated knowledge of how to promote healthy workplaces (Table 4 and Figure 4). Specifically, 14 participants demonstrated an increase, 4 participants exhibited a decrease and 15 participants showed no change (*p* = 0.012, *z* = 2.502, *r* = 0.44). This improvement increased considerably between T1 and T3, with 14 participants showing an increase, 1 participant showing a decrease and 19 participants showing no change (*p* = 0.001, *z* = 3.214, *r* = 0.55).

## Discussion

A quantitative longitudinal pre- and post-intervention study evaluated the effectiveness of a digital education programme developed for municipal politicians accountable for Swedish elder care, focusing on the organisational and social work environment within that sector. The evaluation was based on self-reported responses from Swedish municipality politicians to a questionnaire at T1, before undertaking a digital education programme (*N = *81), at T2, zero to two weeks after the programme (*n = *35), and at T3, three months after the programme (*n = *35).

### Discussion of the method

This study builds on previous findings from two qualitative interview studies with politicians responsible for elder care (Porter and Muhonen, 2021; Porter, 2022), which identified a knowledge gap regarding the awareness of mandatory organisational and social work environment management, concerning the various professions working within elder care. Module 1 of the educational programme was developed from these research findings and specifically aimed at addressing the identified knowledge gaps.

As stated in the method section, module 1 consisted of six pre-recorded digital lectures, each addressing key aspects of the organisational and social work environments within elder care. The first lecture introduced the concept and importance of organisational and social factors in shaping the work environment. The second focused on the Swedish Work Environment Act and the employer's legal responsibilities. The third addressed mental illness in the workplace, highlighting common challenges in elder care settings. The fourth lecture explored employment and working conditions in a changing elder care sector, with particular attention to what is required for staff to remain motivated and committed to their roles. The fifth examined how to manage differences in multi-ethnic care teams, with a focus on ethnical diversity in everyday practice. Finally, the sixth lecture discussed strategies for preventing and managing mental illness in the workplace, emphasising the leadership role and the balance between organisational demands and employee autonomy. The selection of lectures included in module 1 was made by the research team with the aim of addressing identified knowledge gaps found in previous research on municipality politicians in Sweden accountable for elder care (Porter and Muhonen, 2021; Porter, 2022).

Module 2 was developed based on qualitative interview data collected from professionals working in elder care, including care assistants, assistant nurses, registered nurses, physiotherapists, occupational therapists and first-line managers. These professionals represented both special housing and home care settings. The interviews provided first-hand insights into their experiences of their work and work environment, highlighting everyday challenges and needs. The data were analysed thematically and used to inform the structure and content of the module, ensuring that the educational material was contextually relevant and practically applicable to the realities of elder care work.

To ensure the validity of the digital educational programme including module 1, module 2 and the questionnaire a series of rigours measures were undertaken. Feedback was solicited from the Centre for Work Life Studies (CTA), with which both the project leader (last author) and the first author are affiliated. Further feedback was obtained from representatives of the union organisations corresponding to the key professional groups featured in module 2, and from three municipality politicians who did not participate in the present evaluation study.

All invited reviewers engaged with the digital educational material and completed the questionnaire. Based on the feedback from CTA, minor adjustments were made to the sequence of the films within module 1. No additional revision was deemed necessary, apart from affirmations regarding the relevance and appropriateness of the content in both modules.

The questionnaire was unanimously considered clear, accessible and user-friendly.

The validation of module 2 by the union representatives was particularly significant, as it reinforced the programme's relevance across diverse professional roles. The involvement of researchers, union representatives and the target group of politicians in the validation process ensured that the educational content was both accurate and contextually appropriate.

An additional strength of the study lies in the research team's combined expertise in the elder care and their proficiency in developing educational programmes and questionnaire, supported by the CTA research centre. To further assess the relevance and impact of the digital educational programme, a follow-up qualitative study is planned. This will involve conducting in-depth interviews with a sample of participants from the current study, with the aim of gaining deeper insights into their learning experiences and perceptions of the questionnaire in relation to the overall learning process. A broader evaluation of the programme itself, including its design and effectiveness, is planned for a future publication, where we intend to apply systematic evaluation methods.

However, the single-group pretest–posttest research design used in this study has faced criticism regarding its validity (Marsden and Torgerson, 2012). A key threat identified is maturation, which refers to the natural improvement of learners over time due to increased maturity. The potential impact of maturation grows with the time interval between the pretest and the posttest (Marsden and Torgerson, 2012). Another threat is the test effect, where results (in this case, self-rated knowledge levels) may improve simply because participants remember the questions or become more aware of the areas being tested, which can trigger learning after the initial test. To control for this and accurately determine if knowledge changes are due to the educational intervention rather than the pretest itself, Shadish *et al.* (2002) recommend a study design that includes four groups of participants: pretest and posttest with no intervention, pretest and posttest with the intervention, posttest with no intervention and posttest with the intervention (Shadish *et al.*, 2002). When designing the present study, these threats to validity were considered by the research team. The population, namely, municipality politicians, are inherently engaged in continuous learning as their roles necessitate staying informed about their areas of responsibility. Consequently, it is possible that their knowledge enhancement was not solely attributable to the digital education programme but also to other sources, such as interactions with elder care staff, first-line managers or relatives of individuals receiving home care. Furthermore, the questionnaire itself may have acted as a catalyst for learning. These possibilities are something the researchers have discussed and are aware of. To examine such effects, a future study design should also include control groups to assess if it is possible that maturation and completing the questionnaire contribute to learning.

One could possibly question whether our sample of participants (*N* = 81) is representative of the entire population of municipality politicians with responsibility for elder care. In that case, one could also question the possibility of generalising the results by calculating *p*-values and statistical significances (Hirschauer, 2022). Therefore, to get a deeper understanding of the results, the focus of this study has extended beyond statistical significance to interpreting descriptive measures presented in tables and figures. It is thus important to emphasise not only statistical significance but also descriptive measures, such as effect sizes and differences between observed pretest and posttest values.

The Wilcoxon signed-rank test was deemed appropriate for sampled data, since it is a non-parametric test with minimal assumptions about the underlying distribution of data and designed to compare two matched samples (Blom and Holmquist, 1998). However, the test is a rank test based on calculated differences between observed pretest and posttest values, and one objection to using the test could be that data were on Likert scales, and therefore, cannot be assumed to be interval data. Nevertheless, since “[a] variety of studies have shown that the Likert response format produces empirically interval data at the scale level” (Carifio and Perla, 2008, p. 1150), sampled Likert scale data have been treated as interval and translated into numerical values for further analyses.

### Self-rated knowledge of the work of the key professions

This longitudinal study of municipality politicians responsible for Swedish elder care examined the effects of a digital education programme on their self-rated knowledge of the work of key professions in both special housing and home care settings. The intervention led to substantial and lasting knowledge improvements at T2 and T3 across the professional groups. The knowledge of the work of care assistants, assistant nurses, registered nurses, physiotherapists, occupational therapists and first-line managers showed notable gains between T1 and T2, and T1 and T3. Knowledge levels were largely maintained or increased at T3, indicating the intervention's effectiveness. The comprehension of the work of registered nurses, occupational therapists and first-line managers showed the most substantial improvements, with high effect sizes. Although the knowledge of the work of first-line managers exhibited a reduction in effect size for home care at T3, the overall impact remained substantial. These findings are consistent with previous research indicating that targeted educational programmes can effectively enhance professional knowledge and skills in healthcare settings (Lämås *et al.*, 2021; Bölenius *et al.*, 2017). The sustained knowledge gains observed at T3 highlight the importance of continuous education and training for municipality politicians responsible for elder care, in order to maintain high standards of care.

### Self-rated knowledge of legal responsibility for the work environment

The analysis of the intervention's effects on the knowledge of municipality politicians regarding their accountability for the work environments of key professions in elder care reveals several important findings. The results indicate that the intervention led to substantial improvements in self-rated knowledge across investigated areas. Thus, the intervention showed substantial improvements in self-rated knowledge of legal responsibility for the work environment between T1 and T2. However, no clear changes were observed between T2 and T3, indicating that the knowledge gains were maintained over time. The fact that the results showed improvement as described indicates that the digital education programme may be a way to enhance knowledge of legislated work environment management and overall work content in elder care, on an organisational level. This could be considered a step towards a better understanding of how to create a sustainable social and organisational work environment for employees in elder care. Corin and Björk (2016) mentioned the lack of understanding among politicians on the organisational level regarding anything other than financial objectives and a balanced budget: come what may, it must fit into a balanced budget. Perhaps an enhanced sense of responsibility for work environment essentials could help create a better understanding of what is causing the well-known difficulties of elder care, such as high sick leave numbers and employees wanting to leave the public care sector – not because of the work itself but because of work demands and lack of recourses (National Board of Health and Welfare, 2023; Storm, 2018; Swedish Municipal Workers’ Union, 2023; Swedish Social Insurance Agency, 2024; Szebehely *et al.*, 2017). Those examples relate to an individual level but emanate from an organisational level, where decisions are made about the organisation's structure, principles for work and the values that should underpin the work.

### Self-rated knowledge of causes of mental illness

Participants demonstrated substantial increases in their knowledge of the causes of mental illness between T1 and T2. The absence of clear changes between T2 and T3 suggests that the intervention's effects were sustained. The organisational level, that is, municipality politicians, must, on the one hand, understand how job demands risk producing a harmful and even destructive social and organisational work environment, on an individual level, and, on the other hand, comprehend the importance of enabling resources to counteract the job demands. If first-line managers are to enable the establishment of good work environments, they must have the resources to do so. That is, they have to be able to generate resources for their employees, since work resources can, as mentioned above, interact with and buffer the effect of work demands and hence impede mental health problems in individuals caused by a poor social and organisational work environment (Bakker and Demerouti, 2017; Dollard and Bakker, 2010; Xanthopoulou *et al.*, 2007b). Resources in this case are not only monetary resources, but most of all personal aspects that fulfil psychological needs (Bakker, 2011), such as trust and support. It is therefore important to gain knowledge of what is creating trust and support in a work environmental sense and how this is done.

### Self-rated knowledge of how to promote healthy workplaces

The intervention substantially enhanced participants' self-rated knowledge of how to promote healthy workplaces between T1 and T2, and the absence of clear changes between T2 and T3 indicates that the knowledge improvements were maintained. On an organisational level, the results indicate the possibility to create preconditions to eliminate risks in the work environment and hence promote healthy workplaces, but it is, unfortunately, rare to focus on this level in relation to healthcare employees. As stated by the Swedish Agency for Work Environment Expertise (2023) in their systematic literature review, there is plentiful knowledge from research, but this knowledge is not used. Even if the importance of the engagement at the organisational level is spoken of, the most senior level with monetary decision-making power, that is, the municipality politicians are not mentioned as formally responsible on an organisational level (Swedish Agency for Work Environment Expertise, 2023). Those in charge must have applicable knowledge, and importantly, they must know of the liability inherent in their position. In other words, existing knowledge must be used by those accountable on an organisational level. The evaluated educational programme focuses on the liability of politicians and their responsibility to organisationally make it possible to create healthy workplaces for those who actually are working in day-to-day business. The results indicate that digital education programmes are a way to disseminate existing knowledge to this organisational, accountable level.

## Conclusion

This longitudinal, single-group pre-experimental study evaluated the effect of a digital education programme on the self-rated knowledge of organisational and social work environment within elder care, targeting municipality politicians responsible for Swedish elder care. The results showed substantial and lasting self-rated knowledge improvements across the key professional groups in both special housing and home care settings. Important findings include substantial knowledge gains among municipality politicians regarding the work of care assistants, assistant nurses, registered nurses, physiotherapists, occupational therapists and first-line managers, the knowledge regarding all of those professions exhibiting notable improvements between T1 and T2, and T1 and T3. Knowledge levels were largely maintained or increased at T3, indicating the intervention's effectiveness. The municipality politicians' knowledge of the work of registered nurses, occupational therapists and first-line managers showed the most notable improvements, with high effect sizes. Regarding the work of first-line managers, a slight reduction in effect size was shown for home care at T3, but the overall impact remained substantial. The study also revealed substantial improvements in knowledge regarding accountability for work environments, including legal responsibility, as well as regarding causes of mental illness and the promotion of healthy workplaces. The sustained knowledge gains underscore the effectiveness of targeted educational programmes in enhancing professional knowledge and skills among municipality politicians in healthcare settings. Overall, the results suggest that a digital education programme targeting the organisational level of the municipality-driven elder care, that is, accountable politicians can help improve the understanding of work environment management regarding elder care, and thereby be a way to address challenges in elder care employee situations.
